# The contralateral effects of foam rolling on range of motion and muscle performance

**DOI:** 10.1007/s00421-023-05142-2

**Published:** 2023-01-25

**Authors:** Andreas Konrad, Masatoshi Nakamura, Konstantin Warneke, Olyvia Donti, Anna Gabriel

**Affiliations:** 1 Institute of Human Movement Science, Sport and Health, Graz University, Mozartgasse 14, 8010 Graz, Austria; 2 Faculty of Rehabilitation Sciences, Nishi Kyushu University, Ozaki, Kanzaki, Saga Japan; 3 Institute for Exercise, Sport and Health, Leuphana University, Lüneburg, Germany; 4 Sports Performance Laboratory, School of Physical Education and Sport Science, National and Kapodistrian, University of Athens, Athens, Greece; 5 Professorship for Conservative and Rehabilitative Orthopedics, Technical University of Munich, Munich, Germany

**Keywords:** Self-massage, Myofascial system, Myofascial release, Cross-education, Cross-over effect

## Abstract

A single bout of foam rolling (FR) can acutely increase joint range of motion (ROM) without detrimental effects on subsequent muscle performance. Similarly, long-term FR training can increase ROM, while muscle performance seems to be unaffected. Although the acute and long-term effects of FR on the treated muscle are understood, the impact of FR on the contralateral side is not well known. Therefore, this scoping review aims to summarize the current evidence on the acute and long-term effect of FR on the ipsilateral limb on ROM and muscle performance (i.e., maximum force, rate of force development, jump height) for the contralateral (non-treated) limb. Potential explanatory mechanisms are also discussed. There is evidence that a single bout of FR on the ipsilateral limb increases ROM of the contralateral limb; however, evidence is limited for long-term effects. The most likely mechanism for contralateral ROM increases is a reduced perception of pain. With regard to isolated muscle contractions, no changes in muscle performance (i.e., maximum voluntary isometric contraction, maximum voluntary dynamic contraction) were found in the contralateral limb after a single bout of FR on the ipsilateral limb. Notably, only one study reported large impairments in rate of force development of the contralateral limb following FR on the ipsilateral leg, possibly due to decreased motor unit recruitment. Furthermore, to date there are only two studies examining the long-term FR training of the ipsilateral limb on performance (i.e., maximal strength and jump performance) which reported moderate improvements. Although, trivial to very large changes on a variety of parameters were found in this study, the functional and practical relevance of our findings should be interpreted with caution.

## Introduction

A single session of foam rolling (FR) can increase the range of motion (ROM) of a joint immediately after the exercise (Wilke et al. 2020; Nakamura et al. 2021b), and this increase can last for more than 30 min post-exercise (Monteiro et al. 2018; Kasahara et al. 2022a). With regard to the acute effects of FR on different performance measures, a meta-analysis (Wiewelhove et al. 2019) reported a tendency for improvements (*P* = 0.06) in sprint performance (+ 0.7%; ES = 0.28), but negligible effects in jump or strength performance. The studies reporting that a single bout of FR does neither increase nor decrease performance in the treated body region are supported by another review (Cheatham et al. 2015). In addition, Wiewelhove et al. (2019) showed that a single bout of FR can reduce muscle pain and induce faster recovery.

With regard to the training (i.e., chronic, long-term) effects of FR, a recent meta-analysis reported that FR can increase ROM in the long term (ES = 0.82), but only under specific circumstances, such as being performed for more than 4 weeks and when applied on the quadriceps and hamstrings (but not the triceps surae) (Konrad et al. 2022c). With regard to performance parameters, a recent meta-analysis reported no changes following FR training (ES = 0.294; *p* = 0.281) (Konrad et al. 2022a).

Although, in recent years, the evidence on the effects of FR on various parameters, such as ROM, is growing, to date, there is no clear consensus on the contralateral effects of FR. Such a potential cross-over effect of FR from the treated (i.e., ipsilateral) muscle to the contralateral homologous muscle—as seen after stretching (single session: (Chaouachi et al. 2017); stretch training: (Panidi et al. 2021; Nakamura et al. 2022b)) or strength training (Munn et al. 2004; Carroll et al. 2006; Cirer-Sastre et al. 2017)—could be beneficial for, e.g., the treatment of unilateral injuries, and hence for optimizing sports performance. Since the tissue of the contralateral limb is not stressed during exercising the ipsilateral limb, it can be assumed that the effects on the contralateral limb do not occur due to changes in muscle structure/morphology, but rather due to neurological adaptations. The increase in strength of the contralateral limb after several weeks of unilateral resistance training, for example, is likely explained by an enhancement of motor unit recruitment rather than changes in muscle volume (Narici et al. 1989; Munn et al. 2004). Similarly, in stretch training, the acute and chronic changes in muscle function (i.e., increase in ROM) in the contralateral limb can likely be explained by changes in pain perception (i.e., resistance to stretch) (Behm et al. 2021a; Nakamura et al. 2022b) rather than changes in muscle structure, such as muscle stiffness, as it occurs in the ipsilateral limb (Konrad et al. 2017; Nakamura et al. 2021c).

According to recent meta-analyses, there are no differences between stretching and FR with regard to the effects on acute (Konrad et al. 2022b) and long-term (Konrad et al. 2022c) changes in ROM of the ipsilateral leg. Hence, it may be assumed that, as with stretching (Chaouachi et al. 2017; Panidi et al. 2021), FR also affects the contralateral limb through central neural adaptations.

However, to the best of our knowledge, no study has summarized the current evidence concerning the contralateral effects of FR on ROM and performance parameters. Therefore, the purpose of this scoping review is to provide an overview of the whole scope of the acute and long-term contralateral effects of FR, and to discuss potential mechanisms underlying changes in ROM and performance parameters.

## Methods

Due to the high heterogeneity of the included studies in terms of e.g., muscles tested, FR duration as well as the lack of control groups in those studies a scoping review was conducted. Consequently, this review is based on the recommendations of Munn et al. (2018) with regard to scoping reviews. The aims of such a scoping review are to identify the available evidence and potential knowledge gaps. The electronic literature search was performed in three databases (PubMed, Scopus, and Web of Science) using the following search terms/keywords: (non-local OR unilateral OR contralateral OR ipsilateral OR crossover OR remote) AND (“foam rolling” OR “self-myofascial release” OR “roller massage” OR “foam roller”). There was no time restriction for the publication date of the studies. The date of extraction of all the literature from the databases was 10 August 2022. Any studies investigating the contralateral effects of a single bout of FR (only immediate effects, such as 0–5 min post-intervention) or FR training (≥ 2 weeks) on ROM and muscle performance in any population (e.g., patients, athletes) were included in this review. Only studies in English, German, Greek, and Japanese language were included in this review.

**Fig. 1:**
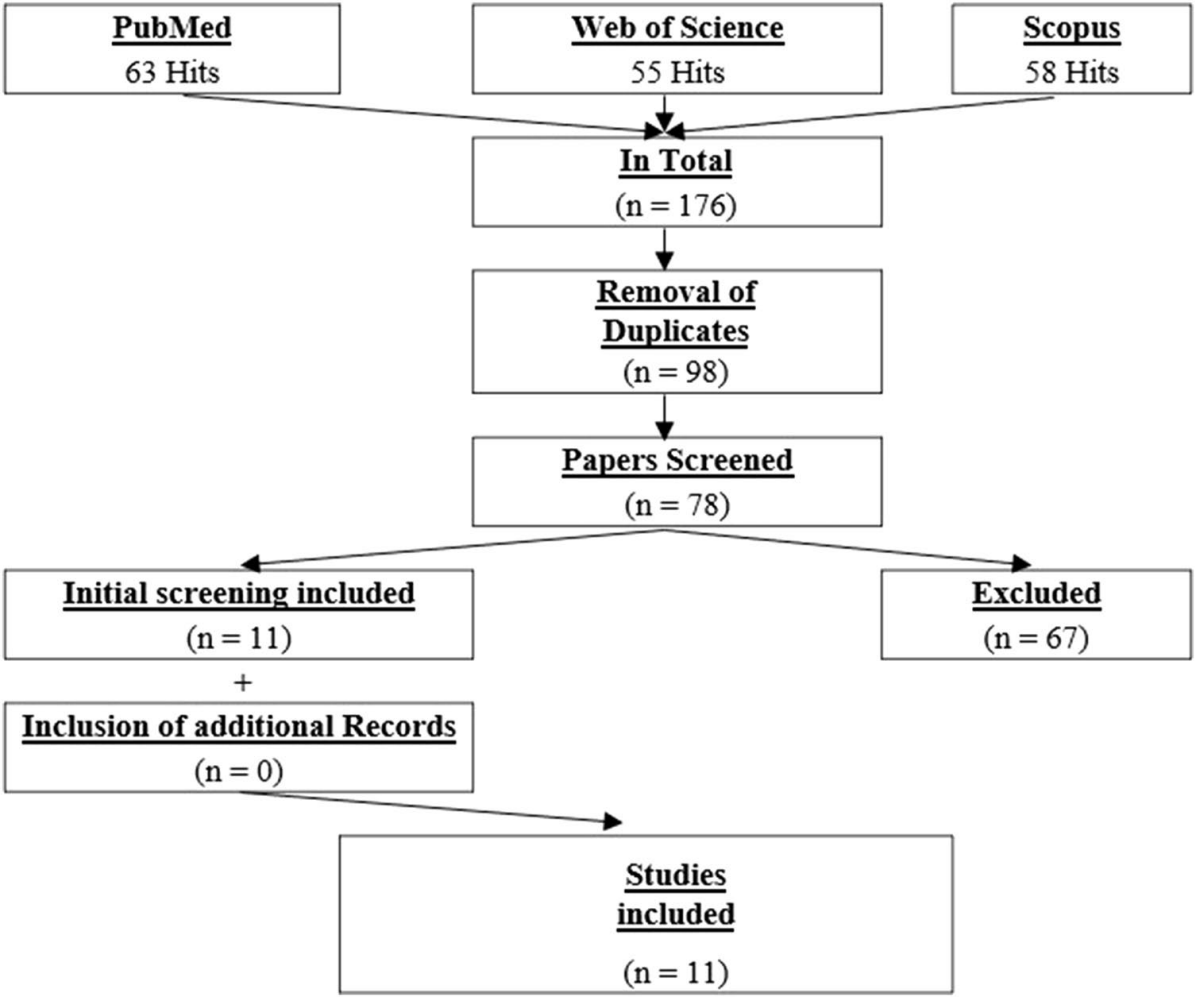
Flowchart of the systematic screening process (PRISMA)

In total, 176 studies were identified. After removing duplicates (*n* = 98), the remaining studies were screened independently by two researchers (AK, MN) by title or, if necessary, by abstract, to identify the studies to be included in this review. Following this blind screening process, the researchers compared their findings and discussed potential mismatches. Overall, 78 studies were screened and 11 met the inclusion criteria. No further eligible studies were detected from the authors’ own libraries or through an additional search of the references of the 11 already included papers. A detailed illustration of the search process is provided in Fig. 1. The characteristics of the included papers are presented in Table 1.

**Table 1:** Participants’ characteristics and specification of the foam rolling intervention of the included studies (*n* = 11)

Study	Participants	Muscle/body area	Design	FR application duration, intensity, details
Acute effects				
Cavanaugh et al. (2017)	12 m; age: 25.1 ± 2.1 years; healthy	Triceps surae muscles	Four groups 1. Ipsilateral FR (stimulated triceps surae muscles, 50 Hz, 400 ms) 2. Contralateral FR 3. Light stroking of the skin with RM on the stimulated triceps surae muscles (sham) 4. Control	3 × 30 s with 30 s rest, VAS 7/10, prone position and passive
Daskalaki et al. (2020)	38f; age: 41.07 ± 5.58 (intervention), 41.18 ± 5.46 (control) years; gym members	Triceps surae muscles	Two groups: 1. Unilateral FR 2. Control group: no FR	3 × 40 s with 20 s rest + 3 × 20 s “hot spot” with 20 s rest + 1 × 40 s with engaged musculature, “tolerable amount of pain”
Kelly and Beardsley (2016)	26 (16 m/10f); age: 24.8 ± 2 (m), 24.4 ± 1.7 (f) years; recreationally active, university students	Triceps surae muscles	Two groups: 1. unilateral FR 2. Control Two measurements: 1. FR leg 2. control leg	3 × 30 s with 10 s rest, slow pace (3 s down and 1 s up), “as much force as possible”, triceps surae muscles on FR with other leg on top and buttocks raised off the floor
Killen et al. (2019)	23 (13 m/10f); age: 27 ± 2 years; healthy	Hamstrings	Two groups: 1. Unilateral FR on dominant leg 2. Static stretching (not reported in this study)	10 × 30 s with 30 s rest, from discomfort (VAS 1/10) to maximal bearable discomfort (VAS 10/10), extended leg with cl leg crossed on top and body weight supported with arms extended on the ground
Nakamura et al. (2021a)	15 (7 m/8f); age: 22.8 ± 3.0 years; healthy	Triceps surae muscles		3 × 60 s with 30 s rest, passive FR with 60 bpm, VAS 7/10, prone position
Ruggieri et al. (2021)^a^	15f; age: 22.9 ± 2.0 years; resistance trained	Hamstrings	Four test sessions: 1. Unilateral FR 2. Unilateral vibrating FR 3. Unilateral vibration-only 4. Control condition	3 × 30 s with 10 s rest, “as much pressure as possible”, 68 Hz vibration, extended leg on FR and ankle of cl leg crossed with weight support of arms behind back
Ye et al. (2019)	36 (25 m/11f) age: 24 ± 4 years; healthy and physically active	Hamstrings	Two groups: 1. FR on dominant leg, cl leg measured 2. Control	10 × 30 s with 30 s rest, from discomfort (VAS 1/10) to maximal bearable discomfort (VAS 10/10), extended leg with cl leg crossed on top and body weight supported with arms extended on the ground
Young et al. (2018)	12 (7 m/5f); age: 26 ± 3 years; healthy	Quadriceps	Four groups: 1. FR on dominant leg 2. Tens 3. FR + Tens 4. Control	4 × 30 s with 30 s rest, VAS 7/10, sitting in special FR device
Yoshimura et al. (2021)	24 (12 m/12f); age: 23.14 ± 2.8 years; healthy	Triceps surae muscles	Two measurements: 1. FR: right leg 2. Control: left leg	3 × 1 min (30 s rest), 15–25% of body weight (measured with force plate), extended leg on FR
Chronic effects				
Hodgson et al. (2018)	23 (13 m/20f); age: 25.1 ± 2.9 (m), 24.9 ± 4.3 (f) years; recreationally active, university students	Quadriceps, hamstrings	Three groups: 1. FR 3 d/week 2. FR 6 d/week 3. control Two measurements: 1. FR leg 2. Control leg	4 weeks, 4 × 30 s alternating for each muscle, sitting with extended leg lying on chair, manual self-FR, 1 s intervals; VAS 7/10
Kasahara et al. 2022b)^a^	30 m; age: 21.6 ± 2.4 years; healthy	Triceps surae muscles	Two groups: 1. Unilateral FR 2. Unilateral vibration FR 2/week, for 6 weeks, with rest period ≥ 48 h between interventions	6 weeks, 2/week, 3 × 60 s with 30 s rest, 60 bpm rolling speed, seated with extended knee on FR, as much pressure as tolerable

*FR* foam rolling or foam roller, *VAS* visual analog scale, *cl* contralateral, *bpm* beats per minute, *RM* roller massage

^a^Studies investigating vibration FR or both FR and vibration FR

The weighted mean percentage changes (pre to post) and the 95% confidence intervals (CIs) (i.e., unweighted means) of the ROM and performance parameters are presented in the subsequent sections. In addition, according to previous suggestions (Hopkins 2004; Behm et al. 2016), we defined the magnitude of the calculated percentage mean changes in the parameters (ROM, performance), i.e., we defined < 0.5%, 0.5% to < 2%, 2% to < 5%, 5% to < 10%, and > 10%, as trivial, small, moderate, large, and very large, respectively.

## Contralateral effects of foam rolling on range of motion

### Acute contralateral effects of foam rolling on range of motion

In total, eight studies investigated the acute and contralateral effects of a single FR exercise on the ROM of various joints. While four studies assessed dorsiflexion ankle ROM after FR on the triceps surae muscles (Kelly and Beardsley 2016; Daskalaki et al. 2020; Nakamura et al. 2021a; Yoshimura et al. 2021), three assessed the ROM of the knee joint after FR on the hamstrings (Ye et al. 2019; Killen et al. 2019; Ruggieri et al. 2021), and one after FR on the quadriceps muscles (Young et al. 2018). Out of these eight studies, nine ROM measures were extracted, since one study analyzed both vibration FR and FR without vibration (Ruggieri et al. 2021) (for more information, see Table 2).

**Table 2:** Summary of the results of the studies which investigated the acute effect and chronic effects of foam rolling on range of motion in the ipsilateral and the contralateral leg

Muscle/body area	Study	Outcome: range of motion		Ipsilateral limb pre-post	Contralateral limb pre-post
Acute effects					
Triceps surae	Daskalaki et al. (2020)	Weight-bearing lunge test		+ 8.41%^a^	+ 3.89%^a^
	Kelly and Beardsley (2016)	Weight-bearing lunge test		+ 8.79%^a^	+ 5.55%^a^
	Nakamura et al. (2021a)	Dorsiflexion ROM + passive torque (dynamometer)		+ 19.66%^a^	+ 13.87%^a^
	Yoshimura et al. (2021)	Passive ankle ROM		+ 6.55%^a^	+ 9.98%
Hamstrings	Killen et al. (2019)	Passive SLR		Not measured	+ 6.87%^a^
	Ruggieri et al. (2021)	Passive SLR	FR	+ 3.38%^a^	+ 1.53%^a^
			Vibration FR	+ 2.64%^a^	+ 1.91%^a^
	Ye et al. (2019)	Passive SLR		Not measured	+ 7.17%^a^
Quadriceps	Young et al. (2018)	Modified Thomas test		+ 3.19%^a^	+ 6.64%^a^
Chronic effects					
Triceps surae	Kasahara et al. (2022b)	Ankle dorsiflexion	FR	+ 16.95%^a^	+ 13.82%^a^
			Vibration FR	+ 31.60%^a^	+ 29.52%^a^
Quadriceps	Hodgson et al. (2018)	Kneeling lunge position, passive knee ROM		3/week FR: − 17.58% 6/week FR: − 0.25%	3/week FR: − 12.22% 6/week FR: − 14.49%
Hamstrings		Passive SLR		3/week FR: + 4.02% 6/week FR: + 1.53%	3/week: + 4.64% 6/week: + 0.54%

+ indicates increase in range of motion (ROM)

− indicates decrease in ROM

*FR* foam rolling, *SLR* straight leg raise test

^a^Significant change between pre and post within the study

Eight out of the nine individual studies investigating ROM measures reported a significant increase (individual results of the studies as a pre-post comparison) in ROM of the contralateral limb following FR exercise on the ipsilateral limb (significant changes marked with a ‘a’ in Table 2).

The mean change of all nine measures included in our analysis shows a large magnitude of improvement of 5.60% (CI (95%) 3.65–8.19%). In particular, the results of FR on the triceps surae muscles (four measures, four studies) showed a large magnitude for the mean increase in contralateral ankle ROM of 5.78% (CI (95%) 3.93–11.39%). Moreover, FR of the hamstrings (four measures, three studies) revealed a large magnitude for mean change in knee extension ROM of 5.26% (CI (95%) 1.63–7.02%) for the contralateral limb. In the future, when more data are available, a meta-analytic approach should be used to test if one muscle group might be more ‘sensitive’ to ROM changes after FR compared to other muscle groups of the contralateral limb. For the ipsilateral limb, Wilke et al. (2020) reported ROM increases after triceps surae (ES = 0.43) and hamstrings FR (ES = 1.0), but no significant changes after quadriceps FR (ES = 0.83), which is in line with the findings of this study. However, the findings in Wilke et al. (2020) were similar to our findings based on only a few effect sizes. Thus these authors concluded that the large variability rather than the ineffectiveness to increase ROM reflected the insignificant results for the quadriceps (Wilke et al. 2020).

Although we found a large increase in ROM in the contralateral limb after a single bout of FR, it should be mentioned that the duration of the FR application varied throughout the included studies (mean: 200 s; S.D. 100.7 s; min: 90 s; max: 340 s). A meta-analysis on stretching reported a tendency of higher increases in ROM of the contralateral limb after > 240 s of stretching (ES = 1.24), compared to < 120 s (ES = 0.72) (Behm et al. 2021a). In the future, when more studies (especially with control groups) are available, the parameter of FR duration should be considered in a meta-analysis, subgroup analysis, or meta-regression analysis.

It has been shown by a recent meta-analysis that the ROM of the ipsilateral limb can be increased after a single bout of FR (Wilke et al. 2020). In addition, further meta-analyses have shown that a single bout of stretching or FR are similarly effective in increasing the ROM of the treated limb acutely (Wilke et al. 2020; Konrad et al. 2022b). The literature on stretching suggests that, after a single bout of ipsilateral limb stretching, the contralateral or even the non-local ROM increases (Chaouachi et al. 2017; Behm et al. 2021b). According to our findings, it is very likely that an FR exercise on the ipsilateral limb can increase the ROM of the contralateral limb. However, it should be mentioned, by comparing the contralateral limb to the ipsilateral limb, that a 3.16% (CI (95%) − 0.65% to 4.77%) more pronounced increase in ROM was found in the ipsilateral limb in our analysis. Consequently, to optimize the acute ROM increase after a single FR exercise, it should be recommended that the FR exercise should be applied on the ipsilateral side when a ROM increase is desired.

### Acute contralateral effects of foam rolling on range of motion in a fatigued muscle

It has been shown that a single FR exercise on a fatigued muscle can restore ROM locally (Nakamura et al. 2020). However, some studies which have explored the effects of an FR intervention of the ipsilateral limb on the contralateral fatigued muscle have reported a beneficial effect on ROM for the ipsilateral limb, compared to the contralateral limb (Jay et al. 2014; Yanaoka et al. 2021). Furthermore, Jay et al. (2014) reported no significant contralateral effect on ROM when comparing pre- to post-muscle fatigue. However, another study reported the beneficial effects of vibration FR on ROM of the contralateral fatigued muscle (Nakamura et al. 2022a). These conflicting results might be due to the high heterogeneity of the methods used (e.g., muscles, FR duration).

### Potential mechanism for the acute changes in range of motion of the contralateral limb

**Fig. 2:**
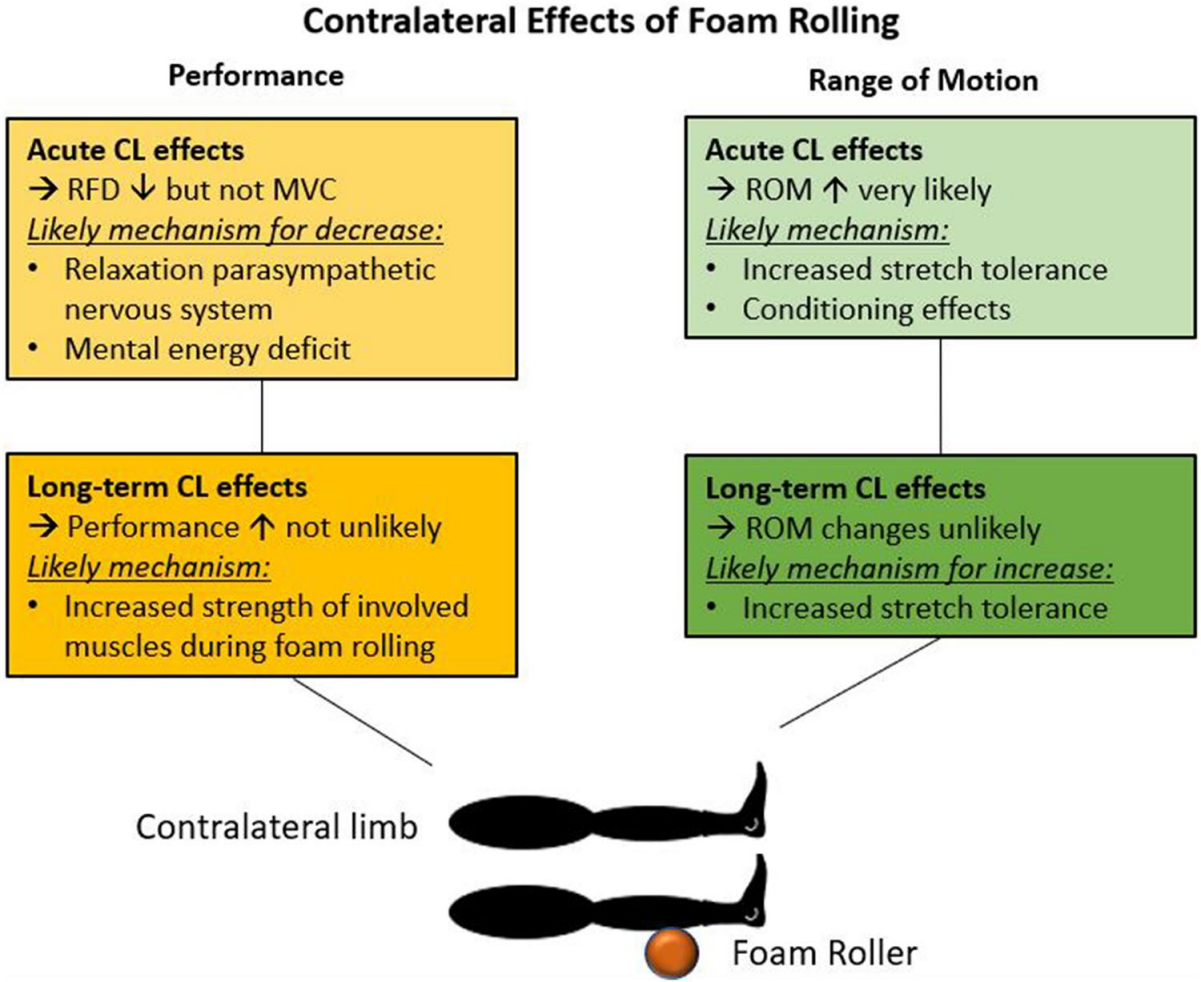
Potential mechanism for the changes in range of motion (ROM) and muscle performance in the contralateral (CL) limb following either a single bout of foam rolling (FR) or FR training on the ipsilateral limb

Considering the mechanisms of ipsilateral ROM increases, it is suggested that, besides increased stretch tolerance, either changes in the muscle structure (i.e., decreased muscle stiffness) or thixotropic effects should be considered as the main mechanism for the increase in ROM after a single bout of FR (Behm and Wilke 2019; Reiner et al. 2021; Konrad et al. 2022b). However, since no mechanical load is applied on the contralateral limb during unilateral FR, changes in the muscle structure (i.e., muscle stiffness) or thixotropic effects are unlikely. Consequently, most of the studies on this topic have suggested changes in stretch tolerance as the main mechanism for the contralateral ROM increases following unilateral FR (Ye et al. 2019; Killen et al. 2019; Daskalaki et al. 2020). This hypothesis was confirmed by a study which reported that the increase in ROM of the contralateral ankle following FR was due to an increased tolerance of pain (i.e., higher tolerated torque during dorsiflexion), and not due to changes in muscle stiffness or spinal excitability (Nakamura et al. 2021a). Furthermore, various other studies have reported higher tolerated pain measured via pressure pain threshold or visual analog scale after FR on the contralateral limb (Aboodarda et al. 2015; Cavanaugh et al. 2017; Cheatham and Baker 2017; Cheatham and Kolber 2018; Nakamura et al. 2021a; Yoshimura et al. 2021), with no significant difference with the ipsilateral limb in pain tolerance (Aboodarda et al. 2015; Cheatham and Baker 2017; Cheatham and Kolber 2018; Nakamura et al. 2021a). Consequently, adjusted perception of pain, either on a global level and/or on the contralateral homologous muscle, can be suggested as the main mechanism for the increased ROM in the contralateral limb. A graphical illustration of the main effects and potential mechanism is provided in Fig. 2.

### Long-term contralateral effects of foam rolling on range of motion

With regard to the long-term effects of FR in the loaded (i.e., ipsilateral) limb, a recent meta-analysis reported that an FR intervention can increase the ROM of the associated joint in the long term, and especially if FR is performed for > 4 weeks and on the thigh muscles (Konrad et al. 2022c). The authors also reported no significant difference between FR and stretching training effects on ROM. Since stretching training for several weeks can also induce contralateral effects on ROM (Panidi et al. 2021; Nakamura et al. 2022b), mainly through altered pain perception, it can be assumed that similar changes will occur in the contralateral limb following FR.

However, according to our systematic search, to date, only two studies have investigated the long-term (i.e., training) effects of FR of the ipsilateral limb on the contralateral limb (see Table 2). Kasahara et al. (2022b) performed a 6-week FR intervention for three times a week with 3 × 60 s bouts of FR on the triceps surae muscles. They randomly assigned the participants to either a conventional or a vibration FR intervention. The results showed similar significant increases in ankle dorsiflexion ROM after the FR interventions in both the ipsilateral and contralateral limbs (see Table 2). Moreover, it should be noted that there was no significant difference between the effects of the conventional and the vibration FR (Kasahara et al. 2022b). A further study performed a four-week unilateral FR training of the quadriceps and hamstrings, either three or six times a week, where each training consisted of 4 × 30 s of FR (Hodgson et al. 2018). Although no interaction effect was reported by the authors, the passive ROM for the quadriceps showed a tendency toward a decrease in both the ipsilateral as well as the contralateral limb. Controversially, for the hamstrings, a tendency of an increase in ROM was reported (see Table 2) (Hodgson et al. 2018).

With regard to the mechanism for an increase in ROM in the contralateral leg after FR training for several weeks, altered perception of pain or stretch is also likely. This was confirmed by the study of Kasahara et al. (2022a, b), who reported a higher tolerated torque at the end ROM (i.e., stretch tolerance), but no changes in muscle stiffness. In contrast, Hodgson et al. (2018) reported no significant changes in ROM, and consequently no changes in the pressure pain threshold of the hamstrings and quadriceps muscles. According to the current evidence, it can be suggested that, after FR training, a contralateral increase in ROM is associated with increased pain tolerance (see Fig. 2).

## Contralateral effects of foam rolling on performance parameters

### Acute contralateral effects of foam rolling on performance parameters

In total, four studies (Cavanaugh et al. 2017; Ye et al. 2019; Killen et al. 2019; Ruggieri et al. 2021) investigated the acute effects of a single FR exercise on the contralateral performance parameters, such as maximum voluntary isometric contraction (MVIC), maximum voluntary dynamic contraction (MVDC), or rate of force development (RFD) of either the triceps surae or hamstrings. Out of these four studies, eight performance measures were extracted (see Table 3).

**Table 3:** Summary of the results of the studies which investigated the acute effect and chronic effects of foam rolling on performance in the ipsilateral and the contralateral leg

Muscle/body area	Study	Outcome: performance		Ipsilateral limb pre-post	Contralateral limb pre-post
Acute effects					
Triceps surae	Cavanaugh et al. (2017)	Plantar flexor MVIC		− 5.16%	− 1.25%
Hamstrings	Killen et al. (2019)	Knee flexion MVIC		Not measured	+ 3.68%
	Ye et al. (2019)	Knee flexion MVIC		Not measured	No significant change—raw data not reported
		RFD 0–50 ms		Not measured	− 31.2%^a^
		RFD 0–100 ms		Not measured	− 16.8%
		RFD 0–200 ms		Not measured	− 10.1%
	Ruggieri et al. (2021)	Knee flexion MVDC	FR	− 9.94%^a^	− 3.34%^a^
			Vibration FR	− 5.44%^a^	− 1.13%^a^
Chronic effects					
Whole body	Hodgson et al. (2018)	Countermovement jump height		3/week FR: + 6.89% 6/week FR: + 6.83%	3/week FR: + 2.01% 6/week FR: + 7.66%
Quadriceps		Knee extension MVIC		3/week FR: + 6.03% 6/week FR: + 3.17%	3/week: + 6.35% 6/week: + 2.50%
Hamstrings		Knee flexion MVIC		3/week FR: − 2.89% 6/week FR: − 7.68%	3/week FR: + 2.51% 6/week FR: − 3.84%
Triceps surae	Kasahara et al. (2022b)	Plantar flexor MVIC	FR	+ 2.40%	+ 0.64%
			Vibration FR	+ 3.80%	+ 2.02%

+ indicates improvement in performance

− indicates decrease in performance

*FR* foam rolling, *MVIC* maximum voluntary isometric contraction, *MVDC* maximum voluntary dynamic contraction, *RFD* rate of force development

^a^Significant change between pre and post within the study

The results of the individual studies indicate that three out of the eight measures showed a significant decrease in performance (individual results of the studies as a pre–post comparison) in the contralateral limb following a single bout of FR on the ipsilateral limb (marked with a ‘a’ in Table 3).

Out of these eight measures, pre-to-post changes were only reported for seven measures. Consequently, the mean change from these seven measures, which were included in our analysis, was a very large magnitude of impairment of − 12.08% (CI (95%) − 18.50% to − 1.62%). However, it should be noted that the main driver for this large magnitude of change was the three RFD measures from a single study (Ye et al. 2019), which ranged from a decrease of − 31.2% (RFD 0–50 ms) to − 10.1% (RFD 0–200 ms). Thus, when only considering MVIC and MVDC (four measures), in contrast, there was almost no change (0.04% (CI (95%) − 2.79% to 2.45%)). Since these analyses were based only on a small sample size and the reports are conflicting, conclusions should be taken with caution.

According to the results of a previous meta-analysis of FR on the ipsilateral limb (Wiewelhove et al. 2019), a decrease in performance in the contralateral limb would not have been expected. Wiewelhove et al. (2019) even reported a tendency of a small improvement in sprint performance after a single bout of FR on the lower leg muscles. However, it was not possible to investigate the contralateral effects of FR on sprint performance and to compare our findings with the findings of Wiewelhove et al. (2019). The authors also reported negligible effects in jump and strength performance. Similarly, we only found a small decrease in strength (i.e., MVIC and MVDC), but this was based on a small sample size.

When considering unilateral stretching, one study reported that, besides the decrease in strength of the ipsilateral limb (− 6.7%; ES = 0.35), strength also decreased in the contralateral limb (− 4.0%; ES = 0.22), but with a small magnitude of change (Behm et al. 2021c). Since a favorable warm-up effect of FR over stretching on performance parameters exists, at least for the ipsilateral limb (Konrad et al. 2021), the decrease after stretching in the contralateral limb (Behm et al. 2021c) and the almost no change in performance in the contralateral limb after FR seen in our analysis seems to be coherent with the stretching literature.

However, it has to be noted that the four included studies varied in terms of FR duration (mean: 195 s; S.D. 121.2 s; min: 90 s; max: 300 s). According to the assumption of a dose–response effect, especially in stretching (Kay and Blazevich 2012; Behm et al. 2015, 2021d), it can be assumed that those studies which performed a very high volume as a warm-up (10 × 30 s Ye et al. 2019; Killen et al. 2019)), would have reported a decrease in performance compared to the lower-volume warm-up (3 × 30 s Cavanaugh et al. 2017; Ruggieri et al. 2021)). Although the one high-volume study reported a decrease in RFD parameters (but not in MVIC) (Ye et al. 2019), the other study even reported a tendency for an increase in performance in the contralateral limb (Killen et al. 2019). Thus, a dose–response effect, as seen in stretching of the ipsilateral limb (Kay and Blazevich 2012; Behm et al. 2015, 2021d), might not occur in the contralateral limb after FR. Indeed, no such dose–response effect after FR on the ipsilateral limb on performance parameters was reported in the plantar flexion MVIC when comparing FR durations of 1 × 30 s vs. 3 × 30 s vs. 10 × 30 s (Nakamura et al. 2021b).

Moreover, not much evidence is available concerning FR of the contralateral fatigued muscle. Although it has been shown that FR on a fatigued muscle can restore muscle performance locally (Kaya et al. 2020; Nakamura et al. 2020), no such effects have been detected on the contralateral fatigued muscle (Nakamura et al. 2022a).

The included studies either reported no change or a decrease in performance in the contralateral limb after unilateral FR. A potential mechanism for such a decrease in performance in the contralateral limb must be related to the central nervous system, since no stimulus happens on the tissue locally. It is likely that, due to the decrease in pain perception of the homologous contralateral muscle (Nakamura et al. 2021a), a further relaxation in the parasympathetic nervous system might happen (Behm and Wilke 2019), which would potentially result in a lower recruitment of motor units. Additionally, a further study suggested that an ipsilateral fatiguing exercise can decrease the mental energy (e.g., focus or concentration) of the subsequent task on the contralateral side (Halperin et al. 2015) (see Fig. 2).

### Long-term contralateral effects of foam rolling on performance parameters

Our systematic search of the training effects of FR on the contralateral limb resulted in the same two eligible studies (Hodgson et al. 2018; Kasahara et al. 2022b), as reported in Sect. 3.2 (i.e., long-term effects on ROM). In total, eight performance measures (countermovement jump height and MVICs) for the contralateral limb were presented by these two studies (see Table 3). Although no significant changes were reported in the individual studies, a mean increase of 2.31% (CI (95%) 0.19–4.74%) was seen for these eight measures in the contralateral limb and a similar increase was seen in the ipsilateral limb (2.15% (CI (95%) − 1.45% to 5.33%)). With regard to the ipsilateral limb, it should be noted that a recent meta-analysis reported no change in performance parameters after FR training over several weeks, based on eight studies (Konrad et al. 2022a). Thus, it can be assumed that there will likely be no long-term effect on performance in the contralateral limb as well.

However, the potential increase in performance in the contralateral limb, which has been shown by our analysis, was likely not based on the FR stimulus itself, but might have resulted from the plank position participants performed while rolling the triceps surae muscles (Kasahara et al. 2022b) or the thigh muscles (Hodgson et al. 2018). Zahiri et al. (2022) showed that the muscle activity (i.e., electromyography) of the core muscles during a plank or reverse plank position is similar to that with quadriceps and hamstrings FR, respectively. Prior studies have also reported co-activation of the lumbar/core and the dorsal thigh muscles, which are linked via connective tissue (Wilke et al. 2016; Krause et al. 2016), during frequently used physical tests aimed at mainly targeting the core muscles (Demoulin et al. 2006; Champagne et al. 2008; Gabriel et al. 2022). Consequently, enhanced core strength might have led to an increase in MVIC in the lower limb and jump performance in the studies of Kasahara et al. (2022a, b) and Hodgson et al. (2018) (see Fig. 2).

## Discussion and conclusion

This scoping review summarized the available literature on the acute and long-term contralateral effects of FR on ROM and performance parameters. While there is evidence that a single bout of FR on the ipsilateral limb will likely increase the ROM of the contralateral limb (e.g., Killen et al. 2019; Daskalaki et al. 2020)), the long-term effects are not well known (Hodgson et al. 2018; Kasahara et al. 2022b). A potential mechanism for the increase in ROM of the contralateral limb, especially following a single bout of FR, could be an increase in non-local and/or contralateral pain tolerance (e.g., Aboodarda et al. 2015; Nakamura et al. 2021a)). With regard to isolated and controlled muscle contractions measured with a dynamometer (MVIC, MVDC), no changes in muscle performance can be expected in the contralateral limb after a single bout of FR on the ipsilateral limb (e.g., Cavanaugh et al. 2017; Killen et al. 2019)). However, one study on RFD reported a big impairment in performance (Ye et al. 2019), which might be based on a relaxation in the parasympathetic nervous system (Behm and Wilke 2019) and hence results in a lower recruitment of motor units. Over the long term, FR on the ipsilateral limb might increase performance (Hodgson et al. 2018; Kasahara et al. 2022b), but this is very likely to be linked to the repeated strength training when performing FR on the posterior or anterior chain of the lower leg muscles in a plank position (Zahiri et al. 2022).

Although, we have found changes in ROM and muscle performance in both acute and long-term FR interventions in the contralateral limb, caution should be taken not to overemphasize the results. Thus, the functional as well as practical relevance of our findings can be debated.

The main limitation of the current evidence is that the included studies investigated the contralateral effects of FR on the leg muscles only, while to date, to the best of our knowledge, there is no evidence for the upper limbs. It is likely that a contralateral effect in the lower limbs on ROM, as reported by Kasahara et al. (2022a, b), only occurs since the contralateral side of the lower limb muscles aims to compensate for the ROM increase of the ipsilateral side during daily life tasks such as walking. Hence, future studies should emphasize exploring muscles which are not that frequently used in daily life, such as the upper limb muscles.

Moreover, future studies should investigate the contralateral effects of FR on the various parameters by including a control group. This would allow a meta-analytic approach to be performed in future reviews. Subsequently, moderator analyses within a meta-analysis could give more insights as to which parameters, such as the various muscles, FR duration, or type of FR (e.g., vibration FR vs. conventional FR), are crucial for potential contralateral effects. With regard to the long-term effects of FR, high-volume durations (e.g., > 30 min a week for > 8 weeks) should be investigated, as it has been seen in stretching studies that sustainable contralateral effects can occur with such a high-volume approach (Panidi et al. 2021).

## Data Availability

The original contributions presented in the study are included in the article. Further inquiries can be directed to the corresponding author.

## Abbreviations

CI: Confidence intervals
ES: Effect size
FR: Foam rolling
MVDC: Maximum voluntary dynamic contraction
MVIC: Maximum voluntary isometric contraction
RFD: Rate of force development
ROM: Range of motion

## References

[CR1] Aboodarda S, Spence A, Button DC (2015). Pain pressure threshold of a muscle tender spot increases following local and non-local rolling massage. BMC Musculoskelet Disord.

[CR2] Behm DG, Wilke J (2019). Do self-myofascial release devices release myofascia? Rolling mechanisms: a narrative review. Sport Med.

[CR3] Behm DG, Blazevich AJ, Kay AD, McHugh M (2015). Acute effects of muscle stretching on physical performance, range of motion, and injury incidence in healthy active individuals: a systematic review. Appl Physiol Nutr Metab.

[CR4] Behm DG, Blazevich AJ, Kay AD, McHugh M (2016). Acute effects of muscle stretching on physical performance, range of motion, and injury incidence in healthy active individuals: a systematic review. Appl Physiol Nutr Metab.

[CR5] Behm DG, Alizadeh S, Anvar SH (2021). Non-local acute passive stretching effects on range of motion in healthy adults: a systematic review with meta-analysis. Sport Med.

[CR6] Behm DG, Alizadeh S, Anvar SH (2021). Non-local acute passive stretching effects on range of motion in healthy adults: a systematic review with meta-analysis. Sport Med.

[CR7] Behm DG, Alizadeh S, Drury B (2021). Non-local acute stretching effects on strength performance in healthy young adults. Eur J Appl Physiol.

[CR8] Behm DG, Kay AD, Trajano GS, Blazevich AJ (2021). Mechanisms underlying performance impairments following prolonged static stretching without a comprehensive warm-up. Eur J Appl Physiol.

[CR9] Carroll TJ, Herbert RD, Munn J (2006). Contralateral effects of unilateral strength training: evidence and possible mechanisms. J Appl Physiol.

[CR10] Cavanaugh MT, Döweling A, Young JD (2017). An acute session of roller massage prolongs voluntary torque development and diminishes evoked pain. Eur J Appl Physiol.

[CR11] Champagne A, Descarreaux M, Lafond D (2008). Back and hip extensor muscles fatigue in healthy subjects: task-dependency effect of two variants of the Sorensen test. Eur Spine J.

[CR12] Chaouachi A, Padulo J, Kasmi S (2017). Unilateral static and dynamic hamstrings stretching increases contralateral hip flexion range of motion. Clin Physiol Funct Imaging.

[CR13] Cheatham SW, Baker R (2017). Differences in pressure pain threshold among men and women after foam rolling. J Bodyw Mov Ther.

[CR14] Cheatham SW, Kolber MJ (2018). Does roller massage with a foam roll change pressure pain threshold of the ipsilateral lower extremity antagonist and contralateral muscle groups? An exploratory study. J Sport Rehabil.

[CR15] Cheatham SW, Kolber MJ, Cain M, Lee M (2015). The effects of self-myofascial release using a foam roll or roller massager on joint range of motion, muscle recovery, and performance: a systematic review. Int J Sports Phys Ther.

[CR16] Cirer-Sastre R, Beltrán-Garrido JV, Corbi F (2017). Contralateral effects after unilateral strength training: a meta-analysis comparing training loads. J Sports Sci Med.

[CR17] Daskalaki K, Pafis G, Gioftsidou A, et al (2020) Investigation of the effects of leg dominance on cross-transfer of flexibility after a unilateral treatment with foam roller-a pilot study. Int J Hum Mov Sport Sci 8:79–85. 10.13189/SAJ.2020.080301

[CR18] Demoulin C, Vanderthommen M, Duysens C, Crielaard JM (2006). Spinal muscle evaluation using the Sorensen test: a critical appraisal of the literature. Jt Bone Spine.

[CR19] Gabriel A, Paternoster FK, Konrad A, et al (2022) Comparison between the original- and a standardized version of a physical assessment test for the dorsal chain—a cohort-based cross sectional study. J Sports Sci Med 21:182–190. 10.52082/JSSM.2022.182 10.52082/jssm.2022.182 PMC9157515 35719223

[CR20] Halperin I, Chapman DW, Behm DG (2015). Non-local muscle fatigue: effects and possible mechanisms. Eur J Appl Physiol.

[CR21] Hodgson DD, Lima CD, Low JL, Behm DG (2018) Four weeks of roller massage training did not impact range of motion, pain pressure threshold, voluntary contractile properties or jump performance. Int J Sports Phys Ther 13:835–845. 10.26603/IJSPT20180835 PMC6159503 30276016

[CR22] Hopkins WG (2004). How to interpret changes in an athletic performance test. Sportsci.

[CR23] Jay K, Sundstrup E, Søndergaard SD (2014). Specific and cross over effects of massage for muscle soreness: randomized controlled trial. Int J Sports Phys Ther.

[CR24] Kasahara K, Konrad A, Yoshida R (2022). Comparison of the prolonged effects of foam rolling and vibration foam rolling interventions on passive properties of knee extensors. J Sport Sci Med.

[CR25] Kasahara K, Konrad A, Yoshida R (2022). Comparison between 6-week foam rolling intervention program with and without vibration on rolling and non-rolling sides. Eur J Appl Physiol.

[CR26] Kay AD, Blazevich AJ (2012). Effect of acute static stretch on maximal muscle performance: a systematic review. Med Sci Sport Exerc.

[CR27] Kaya S, Cug M, Behm DG (2020). Foam rolling during a simulated half-time attenuates subsequent soccer-specific performance decrements. J Bodyw Mov Ther.

[CR28] Kelly S, Beardsley C (2016). Specific and cross-over effects of foam rolling on ankle dorsiflexion range of motion. Int J Sports Phys Ther.

[CR29] Killen BS, Zelizney KL, Ye X (2019). Crossover effects of unilateral static stretching and foam rolling on contralateral hamstring flexibility and strength. J Sport Rehabil.

[CR30] Konrad A, Stafilidis S, Tilp M (2017). Effects of acute static, ballistic, and PNF stretching exercise on the muscle and tendon tissue properties. Scand J Med Sci Sport.

[CR31] Konrad A, Tilp M, Nakamura M (2021). A comparison of the effects of foam rolling and stretching on physical performance. A systematic review and meta-analysis. Front Physiol.

[CR32] Konrad A, Nakamura M, Paternoster FK (2022). A comparison of a single bout of stretching or foam rolling on range of motion in healthy adults. Eur J Appl Physiol.

[CR33] Konrad A, Nakamura M, Tilp M (2022). Foam rolling training effects on range of motion: a systematic review and meta-analysis. Sport Med.

[CR34] Konrad A, Nakamura M, Behm DG (2022). The effects of foam rolling training on performance parameters: a systematic review and meta-analysis including controlled and randomized controlled trials. Inte J Environ Res Public Health.

[CR35] Krause F, Wilke J, Vogt L (2016). Intermuscular force transmission along myofascial chains: a systematic review. Wiley Online Libr.

[CR36] Monteiro ER, Wakefield B, Ribeiro MS (2018). Anterior and posterior thigh self-massage and stretching acutely increases shoulder range-of-motion/Automassagem e alongamento nas regioes anterior e posterior de coxa aumentam de forma aguda a amplitude articular de ombro. Motricidade.

[CR37] Munn J, Herbert RD, Gandevia SC (2004). Contralateral effects of unilateral resistance training: a meta-analysis. J Appl Physiol.

[CR38] Munn Z, Peters MDJ, Stern C, et al (2018) Systematic review or scoping review? Guidance for authors when choosing between a systematic or scoping review approach. BMC Med Res Methodol. 10.1186/s12874-018-0611-x 10.1186/s12874-018-0611-x PMC6245623 30453902

[CR39] Nakamura M, Yasaka K, Kiyono R (2020). The acute effect of foam rolling on eccentrically-induced muscle damage. Int J Environ Res Public Health.

[CR40] Nakamura M, Konrad A, Kiyono R (2021). Local and non-local effects of foam rolling on passive soft tissue properties and spinal excitability. Front Physiol.

[CR41] Nakamura M, Onuma R, Kiyono R (2021). Acute and prolonged effects of different durations of foam rolling on range of motion, muscle stiffness, and muscle strength. J Sport Sci Med.

[CR42] Nakamura M, Yahata K, Sato S (2021). Training and detraining effects following a static stretching program on medial gastrocnemius passive properties. Front Physiol.

[CR43] Nakamura M, Yoshida R, Sato S (2022). Cross-education effect of 4-week high- or low-intensity static stretching intervention programs on passive properties of plantar flexors. J Biomech.

[CR44] Nakamura M, Kasahara K, Yoshida R, Yahata K, Sato S, Murakami Y, Aizawa K, Konrad A (2022). Crosseducationeffect of vibration foam rolling on eccentrically damaged muscles. J Musculoskelet Neuronal Interact.

[CR45] Narici MV, Roi GS, Landoni L (1989). Changes in force, cross-sectional area and neural activation during strength training and detraining of the human quadriceps. Appl J Physiol Occup Physiol.

[CR46] Panidi I, Bogdanis GC, Terzis G (2021). Muscle architectural and functional adaptations following 12-weeks of stretching in adolescent female athletes. Front Physiol.

[CR47] Reiner MM, Glashüttner C, Bernsteiner D (2021). A comparison of foam rolling and vibration foam rolling on the quadriceps muscle function and mechanical properties. Eur J Appl Physiol.

[CR48] Ruggieri RM, Coburn JW, Galpin AJ, Costa PB (2021). Effects of a vibrating foam roller on ipsilateral and contralateral neuromuscular function and the hamstrings-to-quadriceps ratios. Int J Exerc Sci.

[CR49] Wiewelhove T, Döweling A, Schneider C (2019). A meta-analysis of the effects of foam rolling on performance and recovery. Front Physiol.

[CR50] Wilke J, Krause F, Vogt L, Banzer W (2016). What is evidence-based about myofascial chains: a systematic review. Arch Phys Med Rehabil.

[CR51] Wilke J, Müller AL, Giesche F (2020). Acute effects of foam rolling on range of motion in healthy adults: a systematic review with multilevel meta-analysis. Sport Med.

[CR52] Yanaoka T, Yoshimura A, Iwata R (2021). The effect of foam rollers of varying densities on range of motion recovery. J Bodyw Mov Ther.

[CR53] Ye X, Killen BS, Zelizney KL (2019). Unilateral hamstring foam rolling does not impair strength but the rate of force development of the contralateral muscle. PeerJ.

[CR54] Yoshimura A, Sekine Y, Schleip R (2021). The acute mechanism of the self-massage-induced effects of using a foam roller. J Bodyw Mov Ther.

[CR55] Young JD, Spence AJ, Power G, Behm DG (2018). The addition of transcutaneous electrical nerve stimulation with roller massage alone or in combination did not increase pain tolerance or range of motion. J Sport Sci Med.

[CR56] Zahiri A, Alizadeh S, Daneshjoo A (2022). Core muscle activation with foam rolling and static planks. Front Physiol.

